# Transformation Pathway upon Heating of Metastable *β* Titanium Alloy Ti-15Mo Investigated by Neutron Diffraction

**DOI:** 10.3390/ma12213570

**Published:** 2019-10-31

**Authors:** Pavel Zháňal, Petr Harcuba, Josef Stráský, Jana Šmilauerová, Přemysl Beran, Thomas C. Hansen, Hanuš Seiner, Miloš Janeček

**Affiliations:** 1 Department of Physics of Materials, Charles University, Ke Karlovu 5, 12116 Prague, Czech Republic; harcuba.p@gmail.com (P.H.); josef.strasky@gmail.com (J.S.); jana.smilauerova@gmail.com (J.Š.); janecek@met.mff.cuni.cz (M.J.); 2 Material and Mechanical Properties, Research Centre Rez Ltd., Hlavni 130, 25068 Husinec-Rez, Czech Republic; 3 Nuclear Physics Institute v.v.i. ASCR, Hlavni 130, 25068 Husinec-Rez, Czech Republic; pberan@ujf.cas.cz; 4 European Spallation Source ERIC, Box 176, SE-22100 Lund, Sweden; 5 Institut Laue-Langevin, 71 avenue des Martyrs, 38000 Grenoble, France; hansen@ill.fr; 6 Institute of Thermomechanics, ASCR, Dolejskova 5, 18200 Prague, Czech Republic; hseiner@it.cas.cz

**Keywords:** Ti alloys, *α* phase, *β* phase, *ω* phase, phase transitions, neutron diffraction

## Abstract

A transformation pathway during thermal treatment of metastable β Ti-15Mo alloy was investigated by in situ neutron diffraction. The evolution of individual phases α, β, and ω was investigated during linear heating with two heating rates of 1.9 ${}^{\circ }C/\min$ and 5 ${}^{\circ }C/\min$ and during aging at 450 ${}^{\circ }C$. The results showed that with a sufficient heating rate (5 ${}^{\circ }C/\min$ in this case), the ω phase dissolves before the α phase forms. On the other hand, for the slower heating rate of 1.9 ${}^{\circ }C/\min$, a small temperature interval of the coexistence of the α and ω phases was detected. Volume fractions and lattice parameters of all phases were also determined.

## 1. Introduction

Metastable β titanium alloys are mostly used in a wide range of components for the aerospace industry and as alternative structural materials for the automotive sector due to their high specific strength and excellent fatigue resistance [1,2]. This class of titanium alloys is characterized by its ability to retain the high temperature bcc phase during quenching from above the β transus temperature. Due to the metastable nature of the retained β phase, the mechanical properties of metastable β titanium alloys can be tailored through careful control of the microstructure during aging heat treatments. For structural applications that employ metastable β Ti alloys, the homogeneous distribution of fine α precipitates in the β matrix is desirable. Understanding of the kinetics of nucleation and growth of the α phase is therefore of primary importance to be able to control the resulting microstructure and mechanical properties [3,4,5,6].

After quenching from a temperature above the β transus, the microstructure of these alloys often consists of small and densely distributed particles of the athermal ω ($\omega_{ath}$) phase in the metastable β phase matrix. The ω phase forms through consecutive collapse of pairs of ${\left\{111\right\}}_{\beta }$ as a result of a soft phonon mode in the β phase, and the particles are compositionally indiscernible from the surrounding matrix [7,8]. It is widely accepted that these particles can influence the nucleation of the α phase during subsequent heat treatments, thereby providing a method for microstructural control [9]. However, the exact mechanism of α phase formation is still not well understood [10,11,12], and some of the most recent works have suggested that α phase nucleation may, in certain heating regimes, be only indirectly influenced by the ω phase or completely independent of it [6,13,14].

Heat treatment at temperatures between 300 and 500 ${}^{\circ }C$ produces a microstructure consisting of β matrix and particles with a similar crystal structure to that of $\omega_{ath}$, commonly referred to as isothermal ω ($\omega_{iso}$). Coarsening of $\omega_{iso}$ particles is accompanied by a rejection of all alloying elements [15] from ω particles. As these particles offer a potential route for microstructural control, understanding of their evolution is of great interest to the titanium community, which has resulted in numerous studies of the microstructural evolution of different metastable β alloys during different heat treatments [16,17,18,19,20,21,22,23,24,25,26]. All of these studies highlight the need to develop a better understanding of the transformation sequences that occur in metastable β titanium alloys during thermal treatments. Even though there is an obvious interest in studying commercial alloys, their often complex chemical composition makes the interpretation of experimental data very challenging. Consequently, data from simple binary systems can often provide more robust results. In the current work, neutron diffraction (ND) was used to investigate the phase and microstructural evolution in a simple binary metastable β alloy Ti-15Mo in situ during different thermal treatments. Due to coarse grains of the supplied material and the necessity for sufficient statistics of scattered particles, it was necessary to utilize ND, even though for such experiments, X-ray diffraction is often preferred [18].

## 2. Materials and Methods

### 2.1. Materials

In this research, the transformation pathway upon heating of metastable β titanium Ti-15Mo alloy was investigated in situ by ND. Ti-15Mo (15 wt. %, 8.1 at. % of Mo) is used mainly for biomedical applications [27]. It was originally developed for the chemical industry to provide a titanium alloy with improved corrosion resistance, a low elastic modulus, high strength, good fatigue resistance, and good ductility [28]. High temperature applications were also investigated, but thermal handling difficulties and microstructure instability at moderate temperatures prevented wider use in the aerospace industry [29].

The alloy used in this study was prepared by Carpenter Technology Corporation according to the ASTM F2066 standard [30]. All investigated specimens were solution treated above the β-transus temperature (at 900 ${}^{\circ }C$ for 4 h) in a quartz tube filled with high purity Ar and quenched in water. This condition corresponds to the initial state for the investigation. The samples were cylinders with a diameter of 10 mm and a height of 30 mm.

### 2.2. Neutron Diffraction

ND experiments were performed at the Institut Laue-Langevin (ILL), Grenoble, France, at the instrument D20 utilizing neutrons with a wavelength of 1.54 *Å* within the proposal [31]. The detector at D20 covers a scattering range of 153.6 ${}^{\circ }$ and allows diffraction pattern acquisition in the order of seconds as a function of temperature, pressure, or other parameters. Due to this combination of a high incident neutron flux and a large detector solid angle, D20 provides the fastest counting rate, at a given resolution, of any reactor-based neutron diffractometer [32]. The scheme of the instrument can be found in [32,33]. The thermal neutron beam from the reactor first reaches one of its four monochromators. The monochromatic beam hits then the sample, which diffracts it in many directions. The diffracted neutrons are simultaneously counted by the large microstrip multidetector. The counts are accumulated during a certain time to yield a powder diffraction pattern with a suitable intensity [32,33].

ND was measured during three different heat treatments. For the first measurement, linear heating with the heating rate of 1.875 ${}^{\circ }C/\min$ up to 850 ${}^{\circ }C$ was utilized (the best available approximation of the heating rate that was used during the measurement of the evolution of the elastic constants of Ti-5553 in [34]. For simplicity, the slower heating rate will be referred to as 1.9 ${}^{\circ }C/\min$ in the text.). The signal was also acquired during cooling with a rate of 5 ${}^{\circ }C/\min$ down to 160 ${}^{\circ }C$. In the second experiment, a rate of 5 ${}^{\circ }C/\min$ was utilized for both heating and cooling. The maximum temperature was again 850 ${}^{\circ }C$. In the third experiment, the sample was heated up to 450 ${}^{\circ }C$ with a heating rate of 1.9 ${}^{\circ }C/\min$ and aged at this temperature for approximately 7 h.

The acquisition time of a single spectrum was 32 s for a heating rate of 1.9 ${}^{\circ }C/\min$ (one pattern per 1 ${}^{\circ }C$) and 30 s for a heating (cooling) rate of 5 ${}^{\circ }C/\min$. During aging, the acquisition time was set to one minute. In the first measurement (heating with a heating rate of 1.9 ${}^{\circ }C/\min$ followed by cooling with a rate of 5 ${}^{\circ }C/\min$), the sample was placed in a niobium container, which resulted in additional Nb peaks in the diffraction patterns. We realized this fact only after the start of the diffraction experiment, and due to limited allocated time, the measurement had to continue with the Nb container. In the subsequent experiments, a vanadium container, whose nuclei scatter neutrons weakly, was used instead. The whole volume of the specimens was irradiated during the experiments.

Quantitative phase analysis of the diffraction patterns, such as the evolution of the volume fraction of phases and the lattice parameters, was determined by the Rietveld method as implemented in the software Fullprof [35].

### 2.3. Dilatometry

The evolution of the thermal expansion of Ti-15Mo was measured in situ during linear heating with heating rates of 1.9 and 5 ${}^{\circ }C/\min$ up to 850 ${}^{\circ }C$ utilizing the Linseis L75 PT vertical dilatometer. Dilatometry is a technique for characterizing dimensional changes of a material caused by physical or chemical processes. From the dilatometric curve, it is possible to analyze the temperature and kinetics of phase transformations or to calculate the coefficient of thermal expansion. The measured samples were uniform square prisms with dimensions of 20×4×4 $mm^{3}$.

## 3. Results

**Figure 1:**
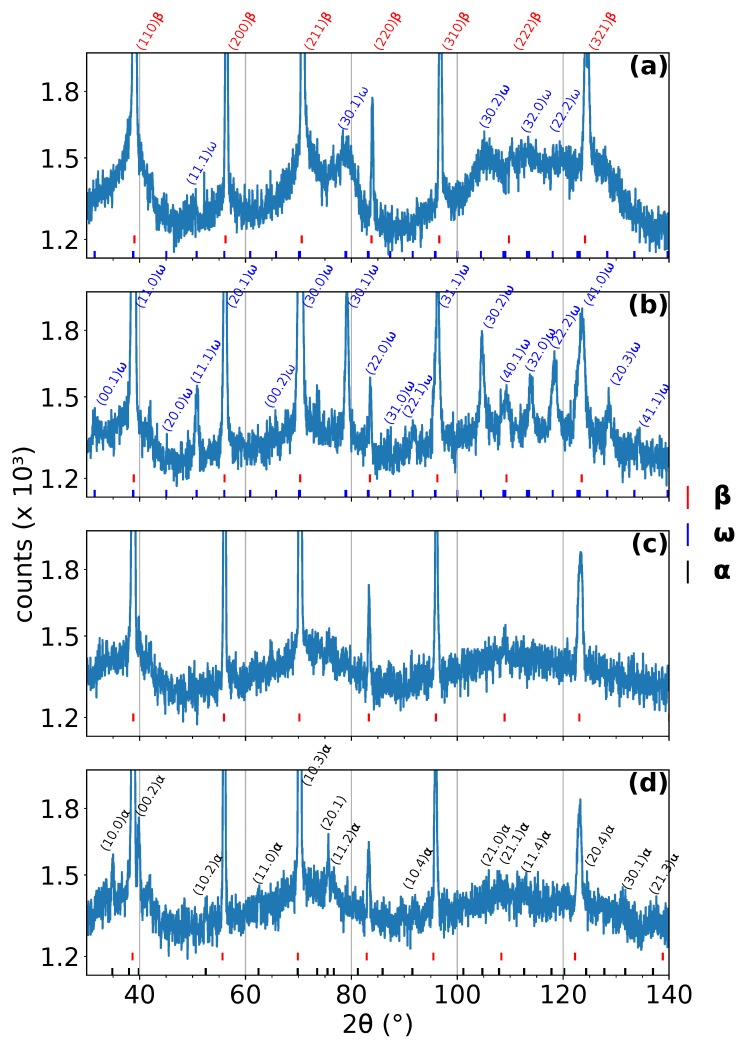
Diffraction patterns acquired at different temperatures during linear heating with a rate of 5 ${}^{\circ }C$/min. (**a**) Room temperature, (**b**) 465 ${}^{\circ }C$, (**c**) 560 ${}^{\circ }C$, and (**d**) 663 ${}^{\circ }C$. β peaks are for clarity indexed only in (**a**). The most distinct ω and α peaks (including those overlapping with β peaks) are indexed in (**b**) and (**d**), respectively.

**Figure 2:**
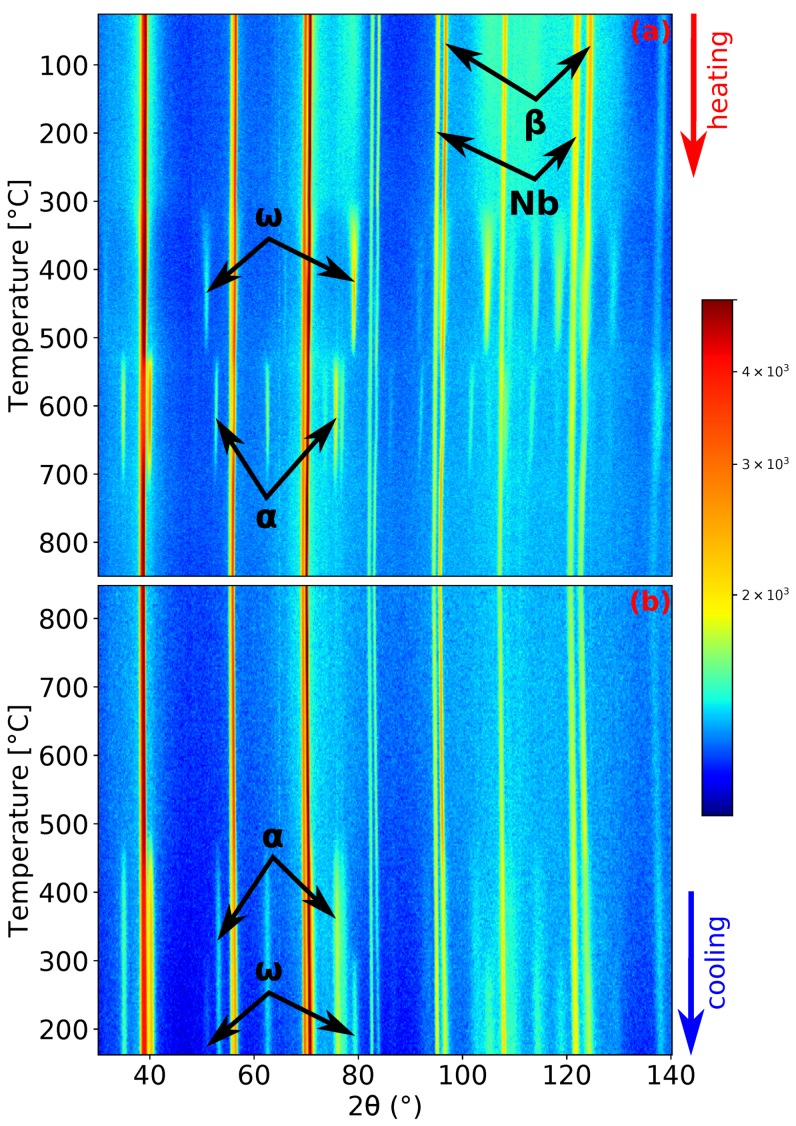
Neutron diffraction (ND) patterns’ evolution of Ti-15Mo during: (**a**) heating with a heating rate of 1.9 ${}^{\circ }C/\min$; (**b**) cooling with a cooling rate of 5 ${}^{\circ }C/\min$.

**Figure 3:**
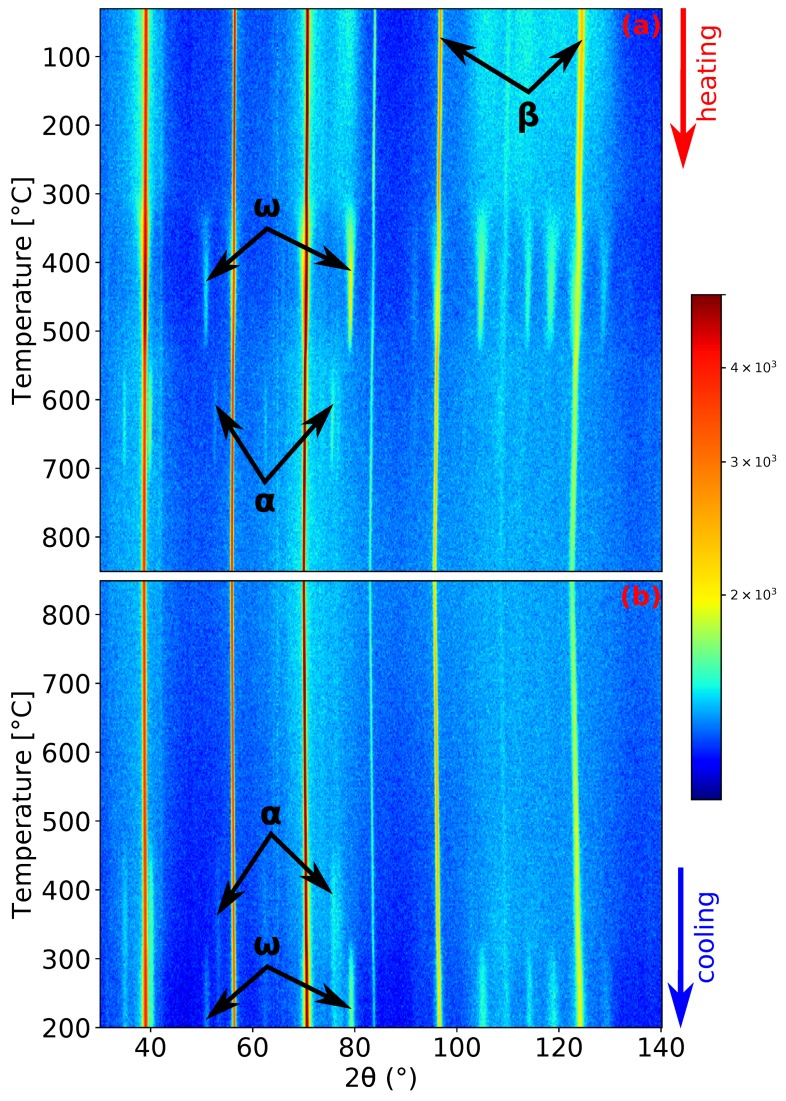
ND patterns’ evolution of Ti-15Mo during: (**a**) heating with a heating rate of 5 ${}^{\circ }C/\min$; (**b**) cooling with a cooling rate of 5 ${}^{\circ }C/\min$.

**Figure 4:**
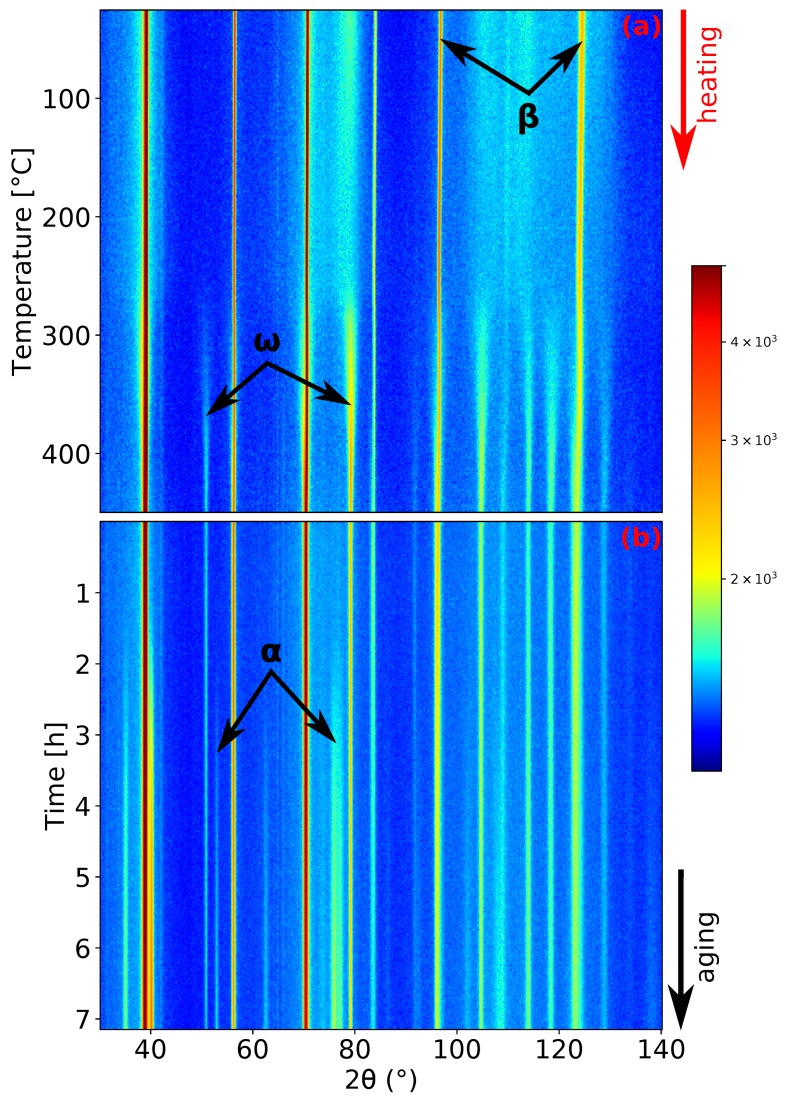
ND patterns’ evolution of Ti-15Mo during: (**a**) heating with the heating rate of 1.9 ${}^{\circ }C/\min$; (**b**) aging at 450 ${}^{\circ }C$.

Four representative diffraction patterns from the measurement with a heating rate of 5 ${}^{\circ }C/\min$ are shown in Figure 1. The diffraction patterns were acquired at: (a) room temperature, (b) 465 ${}^{\circ }C$, (c) 560 ${}^{\circ }C$, and (d) 663 ${}^{\circ }C$. The diffraction patterns in Figure 1b,d correspond to the maximum amplitude of the ω and α phase peaks, respectively. On the other hand, the diffraction pattern in Figure 1c represents the temperature interval, where ω phase has already been completely dissolved and α has not formed yet. The positions of individual peaks of individual phases are shown below each diffraction pattern. The most distinct peaks are indexed. These diffraction patterns serve mainly as examples of high quality data obtained by ND at instrument D20 at ILL; the evolution of the phase composition during ND experiments can be better observed in color coded 2D plots, see Figure 2, Figure 3 and Figure 4. All measured patterns are displayed with the same logarithmic scale, which allows their direct comparison.

Let us now discuss in more detail the kinetics of phase transformations during the slower heating rate of 1.9 ${}^{\circ }C/\min$. ND patterns demonstrating the evolution of Ti-15Mo during heating and cooling are presented in Figure 2. The material at room temperature consists of β phase matrix and ω particles [13], although ω peaks are wide at this temperature (see the ω peaks in Figure 1a) due to the small size of the ω particles [14]. The presence of Nb peaks in Figure 2 is due to the Nb sample holder, as was explained in Section 2. The peaks of the ω phase begin to sharpen during heating around 300 ${}^{\circ }C$, which is caused by coarsening of ω particles. ω peaks reach the maximum intensity at about 450 ${}^{\circ }C$. At higher temperatures, the intensities of the ω peaks decrease. Before the complete dissolution of the ω phase, the α phase starts to form. At the slower heating rate, there was about a 9 ${}^{\circ }C$ interval of the coexistence of the α and ω phases. The α phase peaks reach the maximum intensity at about 633 ${}^{\circ }C$. During subsequent heating, the α phase diffraction maxima intensities decrease and completely disappear around 730 ${}^{\circ }C$, which is consistent with the β transus temperature determined in our previous works by the electrical resistance, dilatometry, and differential scanning calorimetry measurements [13,16,36]. At the highest temperature of 850 ${}^{\circ }C$, the material contains only the β phase. During cooling (see Figure 2b), the α and ω phases form at about 500 and 320 ${}^{\circ }C$, respectively. One can see that the cooling rate of 5 ${}^{\circ }C/\min$ is insufficient to suppress α phase formation. However, the amount of precipitated α phase was not enough to stop ω formation.

The record of the second experiment, heating and cooling with a rate of 5 ${}^{\circ }C/\min$, is shown in Figure 3. The initial condition of the material was the same as in the previous experiment. Due to the faster heating rate and the processes occurring being diffusion controlled, the maxima of the intensity of ω and α peaks are shifted to higher temperatures: 465 ${}^{\circ }C$ and 663 ${}^{\circ }C$ for the ω and α phase (see Figure 1b,d), respectively. Unlike the previous experiment, the α phase did not precipitate before the complete dissolution of ω phase (cf. Figure 1c, where pure β can be observed). The α phase dissolves at a similar temperature as in the previous case (around 730 ${}^{\circ }C$). At the highest reached temperature of 850 ${}^{\circ }C$, the material again consists solely of the β phase. Unsurprisingly, during cooling, the material exhibits the same behavior as in the previous case.

During the last experiment, the evolution of microstructure during aging was studied. The sample was heated up to 450 ${}^{\circ }C$ with a heating rate of 1.9 ${}^{\circ }C/\min$ and then aged at this temperature for about 7 h. This aging temperature was chosen aiming to enhance the coarsening of ω particles, which should further act as preferential nucleation sites for α precipitation. as was reported in [10,37]. The record of this measurement is presented in Figure 4. The α phase forms after approximately 1.5 h of aging (see Figure 4b).

## 4. Discussion

### 4.1. Volume Fraction Evolution

**Figure 5:**
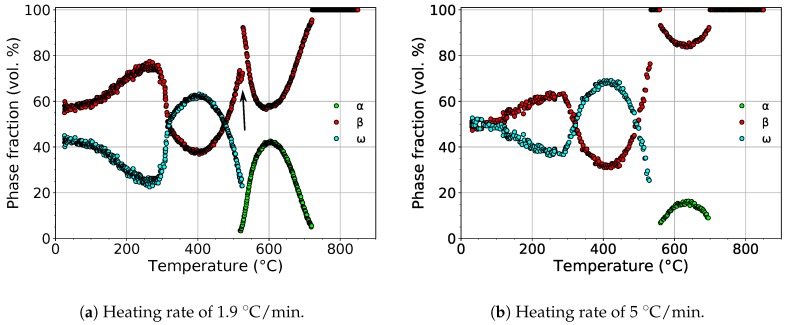
The evolution of the volume fractions of the α, β, and ω phases during heating.

The evolution of the volume fractions of the α, β, and ω phases during heating with heating rates of 1.9 and 5 ${}^{\circ }C/\min$ is shown in Figure 5.

For both heating rates, the volume fraction of the ω phase initially decreased. The scatter of values at low temperatures was due to the extremely small dimensions of ω particles, which disallowed precise fitting of the diffraction patterns. The difference of phase fractions for both heating rates at room temperature was caused by difficult fitting of small and wide ω diffraction maxima. At about 300 ${}^{\circ }C$, the volume fraction of the ω phase started to increase, reaching its maximum at about 400 ${}^{\circ }C$. The calculated maximum volume fraction of the ω phase was 63% and 68% for heating rates of 1.9 and 5 ${}^{\circ }C/\min$, respectively.

During further heating, the volume fraction of the ω phase continuously decreased up to about 550 ${}^{\circ }C$, above which the ω phase completely disappeared. For the slower heating rate, a narrow temperature range (∼9 ${}^{\circ }C$) of the coexistence of the α and ω phases can be observed. This was probably caused by the precipitation of the α phase on grain boundaries [38,39], presumably at the expense of the β phase. This hypothesis was supported by the fact that the volume fraction of the β phase decreased (marked with the black arrow in Figure 5a), while the rate of the decrease of the volume fraction of the ω phase remained constant. The volume fraction of the α phase reached the maximum value of 42% and 15% above 600 ${}^{\circ }C$ for heating rates of 1.9 and 5 ${}^{\circ }C/\min$, respectively. During subsequent heating, the α phase dissolved back to the β phase.

**Figure 6:**
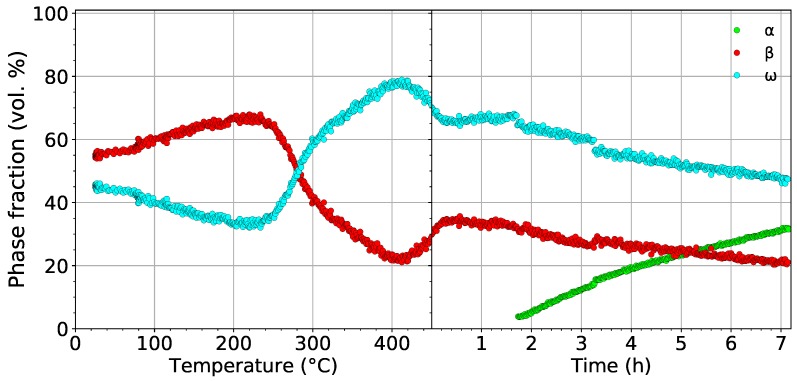
The evolution of the volume fractions of the α, β, and ω phases during heating with a heating rate of 1.9 ${}^{\circ }C/\min$ up to 450 ${}^{\circ }C$ followed by aging for 7 h.

The development of the volume fractions of the α, β, and ω phases in the material aged at 450 ${}^{\circ }C$ is shown in Figure 6. In the left part, the evolution of the β and ω phases during heating to 450 ${}^{\circ }C$ with a rate of 1.9 ${}^{\circ }C/\min$ is shown. The volume fraction of ω phase reaches its maximum value of 77% at 420 ${}^{\circ }C$. The evolution of the volume fraction of the ω phase during heating differs from the first experiment (cf. Figure 5a and Figure 6), despite both the material and the heating rate being the same. This difference is caused by the presence of Nb diffraction maxima in the diffraction patterns from the first measurement. This adverse fact affects fitting of the patterns from the first experiment, which subsequently influences the result. In the case of the presence of Nb peaks, another “phase” must be considered, which adds more parameters to the fitting procedure. Therefore, the results of the second experiment presented in Figure 6, where the Nb container was avoided, are considered more reliable. In the right part of Figure 6, the evolution of volume fractions during aging at 450 ${}^{\circ }C$ is displayed. Small step changes in the evolution of phase fractions after 1.5 h and 3.3 h of aging are caused by the necessary change of fitting parameters and are not caused by any process in the material. The formation of α precipitates, which occurs after about 1.5 h of aging, and their further growth cause a decrease of the volume fractions of both the β and ω phases. After the aging (∼7 h), the volume fractions of the β and ω phases decrease from 35% to 20% and from 65% to 45%, respectively, while the volume fraction of α increases to approximately 35%. The decrease of the volume fraction of the ω phase can be attributed either to α phase growth by consuming ω particles or to the slow dissolution of ω particles back to the β phase. The decrease of the volume fraction of the β phase is obviously caused by the β→α transformation. Due to limited time for the experiment, the sample was quickly cooled down to 150 ${}^{\circ }C$. A final diffraction pattern was acquired (not shown here) at this temperature; the volume fractions of the α, β, and ω phases were determined as 35%, 15%, and 50%, respectively. Note that the volume fraction of the α phase did not change significantly during cooling. On the other hand, the volume fraction of the ω phase increased at the expense of the β phase.

The kinetics of α phase growth during aging at 450 ${}^{\circ }C$ was determined from the Avrami equation [40,41]:

(1) $$\zeta =A\{1-\exp\left[-k{\left(t-\tau \right)}^{n}\right]\},$$

where ζ represents the volume fraction of the nucleated phase, *k* is the growth rate constant, *A* corresponds to the saturation value of the precipitating phase, *t* stands for time, and the exponent *n* corresponds to the conditions of the growth of the newly-formed phase. In the standard Avrami equation, τ refers to the incubation period. However, in this case, it denotes the time needed for the α phase to precipitate since the beginning of aging. Due to the lamellar shape of α precipitates [42], the exponent *n* was set to n=1, which corresponds to diffusion controlled growth of needles or plates [41]. The experimental data were fitted using Equation (1) by the least squares method. The determined coefficients are:

$$\begin{array}{cc} A & =(45\pm 0.4),\\ k & =\left(0.207\pm 0.004\right)\;h^{-1},\\ \tau & =(1.39\pm 0.01)\;h=(83.3\pm 0.6)\;\min. \end{array}$$

**Figure 7:**
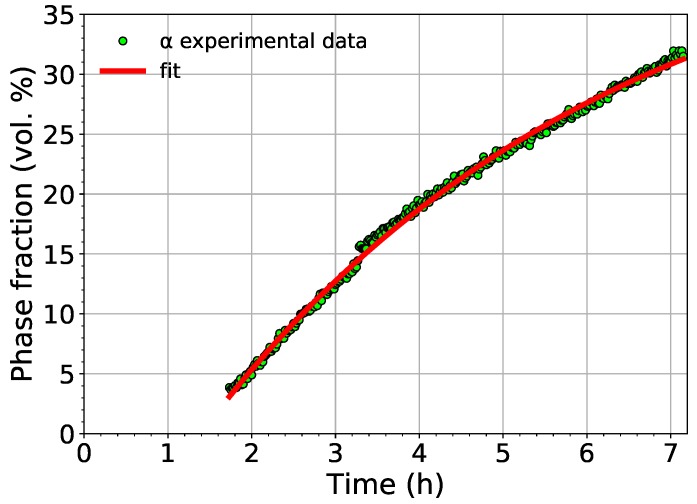
The comparison of the measured volume fraction of the α phase with the fit employing Equation (1).

The comparison of the experimentally determined volume fraction of the α phase with the fit using Equation (1) is shown in Figure 7.

The fit shows that the α phase starts to nucleate in Ti-15Mo after aging for 83 minutes at 450 ${}^{\circ }C$ (the aging time depends obviously on the utilized heating rate). The saturated value of the volume fraction of the α phase was determined to be 45%.

### 4.2. Evolution of Lattice Parameters

**Figure 8:**
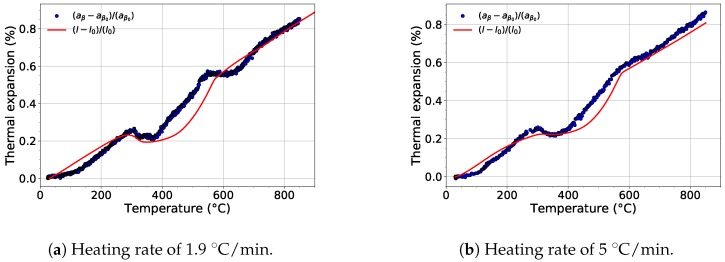
The comparison of the evolution of the lattice parameter of the β phase during heating and relative thermal expansion of Ti-15Mo alloy.

For each experiment, the evolution of the lattice parameters of each phase was determined. Figure 8 shows the comparison of the evolution of the lattice parameter $a_{\beta }$ of the β phase and the thermal expansion of the material during heating with a heating rate of 1.9 and 5 ${}^{\circ }C/\min$. The temperature dependence of the lattice parameter during heating with the slower rate exhibits two deviations from the linear course (Figure 8a). These deviations between 300 and 550 ${}^{\circ }C$ and 550 and 730 ${}^{\circ }C$ are caused by diffusion controlled processes connected to the precipitation of the ω and α phases, respectively. The growth of both the ω and α phases is accompanied by diffusion of Mo into the β phase, which causes $a_{\beta }$ to shorten [43]. After both phases (α, ω) dissolve, i.e., above the β-transus temperature, the evolution of the lattice parameter $a_{\beta }$ returns back to its linear trend.

For the faster heating rate, the first deviation (300–550 ${}^{\circ }C$), which is associated with β↔ω transformation, is well visible (see Figure 8a). On the other hand, the second decrease of $a_{\beta }$ around 700 ${}^{\circ }C$) is much less distinct. This is caused by the much lower volume fraction of the precipitated α phase than for the slower heating rate (cf. Figure 2). When the evolution of $a_{\beta }$ is compared with the results of dilatometry measurements from [16,44,45], it is obvious that the thermal expansion of Ti-15Mo alloy during heating is primarily affected by the evolution of the lattice parameter of the β phase. The absence of the second decrease in the thermal expansion curves is probably caused by the evolution of the lattice parameter of the α phase, which is discussed later.

**Figure 9:**
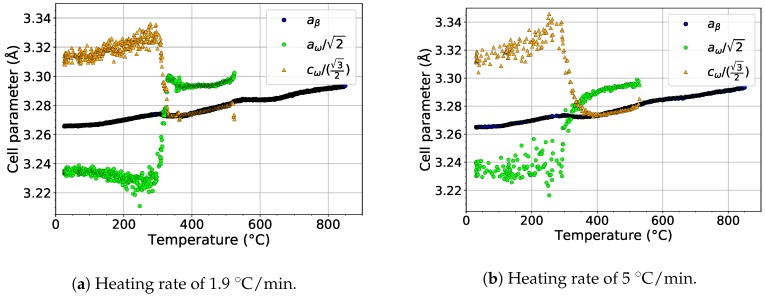
The comparison of the evolution of the lattice parameters of the β and ω phases during heating.

The formation of the ω phase by the collapse of pairs of ${\left\{111\right\}}_{\beta }$ planes and the orientation relationship between β and ω phases [46] (${\left\{0001\right\}}_{\omega }\left\|{\left\{111\right\}}_{\beta },\;{\langle 11\bar{2}0\rangle }_{\omega }\right\|{\langle 011\rangle }_{\beta }$) allows the determination of the size of the ideal lattice parameter of the ω phase as $a_{\omega }=\sqrt{2}a_{\beta }$ and $c_{\omega }=\sqrt{3}/2a_{\beta }$. These relations allow to compare directly the evolution of the lattice parameters of the β ($a_{\beta }$) and ω phases ($a_{\omega }$ and $c_{\omega }$), as shown in Figure 9. The scatter of the values of $a_{\omega }$ and $c_{\omega }$ at temperatures below 300 ${}^{\circ }C$ is due to the extremely small size of ω particles, which makes it difficult to fit the diffraction maxima of the ω phase. For both heating rates, $a_{\omega }$ and $c_{\omega }$ behave similarly. Both parameters exhibit a significant change at about 300 ${}^{\circ }C$. $a_{\omega }$ steeply increases above its ideal value, while $c_{\omega }$ decreases to its exact ideal value. This change is probably connected with the diffusion driven coarsening of ω particles (see the distinct sharpening of ω diffraction maxima around 300 ${}^{\circ }C$ in Figure 2a and Figure 3a for a heating rate of 1.9 and 5 ${}^{\circ }C/\min$, respectively). Due to disappearance of ω particles at about 550 ${}^{\circ }C$, no data for ω lattice parameters are shown above this temperature.

**Figure 10:**
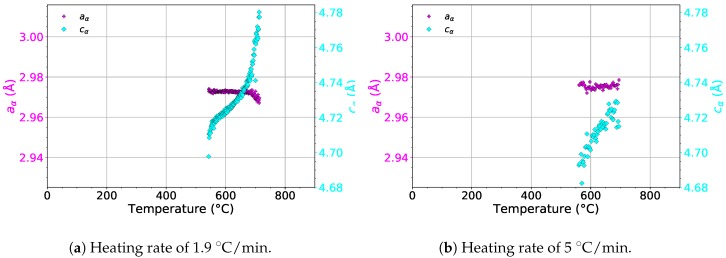
The evolution of the lattice parameters of the α phase during heating.

The evolution of the lattice parameters $a_{\alpha }$ and $c_{\alpha }$ of the α phase during heating with heating rates of 1.9 and 5 ${}^{\circ }C/\min$ is presented in Figure 10. For both heating rates, $a_{\alpha }$ remains almost constant. On the other hand, $c_{\alpha }$ increases in the whole temperature range in the presence of the α phase for both heating rates. The increase of $c_{\alpha }$ probably balances the changes of $a_{\beta }$ in the temperature range of 550–730 ${}^{\circ }C$. As a result, the thermal expansion curve (see the red line in Figure 8a) does not deviate from linearity during α phase precipitation and dissolution, as opposed to the evolution of $a_{\beta }$.

**Figure 11:**
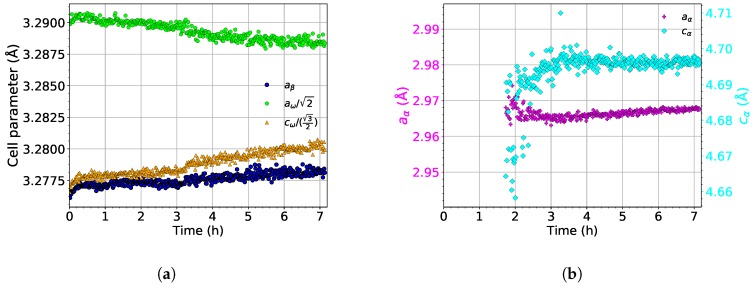
The evolution of the lattice parameters of the (**a**) β and ω and (**b**) α phases during aging at 450 ${}^{\circ }C$.

The evolution of the lattice parameters of the α, β, and ω phases during aging at 450 ${}^{\circ }C$ is shown in Figure 11. As the evolution of lattice parameters during heating up to 450 ${}^{\circ }C$ is the same as during the heating experiment (the same heating rate of 1.9 ${}^{\circ }C/\min$ was used), only their development during aging is presented.

The lattice parameter of the β phase ($a_{\beta }$) slightly increases during the aging process; see the blue dots in Figure 11a. This increase is probably caused by the dissolution of the ω phase, followed by diffusion of Mo into Mo depleted compositional pockets left by dissolved ω particles. Therefore, the average concentration of Mo in the β phase decreases, which should cause a decrease of $a_{\beta }$ [43].

The lattice parameter of the ω phase $c_{\omega }$ slightly increases during the entire aging in a similar manner as $a_{\beta }$ (see Figure 11a). During aging, $c_{\omega }$ slightly deviates from its ideal length. On the other hand, $a_{\omega }$ decreases during aging and approaches its ideal value.

The evolution of the lattice parameters of the α phase ($a_{\alpha }$, $c_{\alpha }$) is shown in Figure 11b. The initial increase of $c_{\alpha }$ is probably an artifact of fitting. The newly nucleated α precipitates are small thin lamellae, disallowing to fit diffraction maxima from these particles precisely. Otherwise, during aging, the lattice parameters of the α phase remain constant.

## 5. Conclusions

Neutron diffraction proved to be an efficient in situ technique for the investigation of the transformation pathway during heating of Ti-15Mo alloy. The volume fractions of the α, β, and ω phases were determined. It was shown that the heating rate influenced the kinetics of the formation/dissolution of individual phases.

The following transformation pathways were observed:

- For the heating rate of 1.9 ${}^{\circ }C/\min$:
  $$\begin{array}{c} \underset{<\;550{\;}^{\circ }C}{\underbrace{\beta +\omega }}⟶\underset{550-560\;{}^{\circ }C}{\underbrace{\beta +\omega +\alpha }}⟶\underset{560-730{\;}^{\circ }C}{\underbrace{\beta +\alpha }}⟶\underset{>\;730{\;}^{\circ }C}{\underbrace{\beta }} \end{array}$$
- For the heating rate of 5 ${}^{\circ }C/\min$:
  $$\underset{<\;550{\;}^{\circ }C}{\underbrace{\beta +\omega }}⟶\underset{550-560\;{}^{\circ }C}{\underbrace{\beta }}⟶\underset{560-730{\;}^{\circ }C}{\underbrace{\beta +\alpha }}⟶\underset{>\;730{\;}^{\circ }C}{\underbrace{\beta }}$$
- For the heating rate of 1.9 ${}^{\circ }C/\min$ and aging at 450 ${}^{\circ }C$ for seven hours:
  $$\beta +\omega \overset{\mathrm{heating}}{\to }\beta +\omega \overset{\mathrm{aging}}{\to }\beta +\omega +\alpha$$

From the presented transformation pathways, it was obvious that with a sufficient heating rate (5 ${}^{\circ }C/\min$ in this case), the ω phase dissolved before the α phase formed. Even for the slower heating rate, we believe that α precipitates did not grow at the expense of ω phase; rather, α grew from the β phase, as a decrease of the volume fraction of the β phase was observed upon the onset of α precipitation.

The material in the initial condition consisted of 50–60% of β phase matrix and about 40–50% of ω particles. The maximum amount of the ω phase of 77% was reached at 420 ${}^{\circ }C$ after heating with a heating rate of 1.9 ${}^{\circ }C/\min$.

It was also shown that the α, β, and ω phases could coexist when a relatively slow cooling rate was employed, which was insufficient to suppress α phase formation. Another possibility to observe α and ω together was by aging the material at a convenient temperature below the stability limit of the ω phase (560 ${}^{\circ }C$).
